# State Entropy and Differentiation Phenomenon

**DOI:** 10.3390/e20060394

**Published:** 2018-05-23

**Authors:** Masanari Asano, Irina Basieva, Emmanuel M. Pothos, Andrei Khrennikov

**Affiliations:** 1 Liberal Arts Division, National Institute of Technology, Tokuyama College, Gakuendai, Shunan, Yamaguchi 745-8585, Japan; 2 Department of Psychology, City University London, London EC1V 0HB, UK; 3 International Center for Mathematical Modeling in Physics and Cognitive Sciences Linnaeus University, 351 95 Växjö-Kalmar, Sweden; 4 National Research University of Information Technologies, Mechanics and Optics, St. Petersburg 197101, Russia

**Keywords:** density operator, state entropy, von Neumann entropy, quantum measurement, differentiation

## Abstract

In the formalism of quantum theory, a state of a system is represented by a *density operator*. Mathematically, a density operator can be decomposed into a weighted sum of (projection) operators representing an ensemble of pure states (a state distribution), but such decomposition is not unique. Various pure states distributions are mathematically described by the same density operator. These distributions are categorized into classical ones obtained from the Schatten decomposition and other, non-classical, ones. In this paper, we define the quantity called the *state entropy*. It can be considered as a generalization of the *von Neumann entropy* evaluating the diversity of states constituting a distribution. Further, we apply the state entropy to the analysis of non-classical states created at the intermediate stages in the process of *quantum measurement*. To do this, we employ the model of *differentiation*, where a system experiences step by step state transitions under the influence of environmental factors. This approach can be used for modeling various natural and mental phenomena: cell’s differentiation, evolution of biological populations, and decision making.

## 1. Introduction

In quantum theory, a state of a system is represented by a density operator. A density operator, e.g., ρ, can be decomposed into a weighted sum of (projection) operators representing “pure states”. This linear combination represents a statistical distribution of pure states in an ensemble of systems. However, the same density operator ρ can be decomposed in various ways. Hence, numerous statistical state distributions are mathematically encoded by the same ρ, unless ρ coincides with a pure state.

One class of these statistical distributions, namely, obtained from “*Schatten decompositions*” of ρ, plays a special role. We remark that, for a density operator with degenerate spectrum, Schatten decomposition is not unique. Any selection of orthogonal bases in eigensubspaces of ρ generates some Schatten decomposition. Each Schatten decomposition corresponds to the statistical distribution of eigenstates of ρ. The crucial point is that these eigenstates may be distinguishable on the basis of measurement of some physical quantity *X*, because these states are orthogonal to each other. The eigenvalues are interpreted as the frequency probabilities of the measurement outcomes. In this sense, the distribution corresponding to the concrete Schatten decomposition of the density operator ρ is conceptually equivalent to a “classical” or “standard” probability distribution.

On the other hand, other decompositions of the same state ρ are “non-classical” or “non-standard” and represent ensembles of pure states which may be not orthogonal to each other. In Section 2, we discuss these points in more detail.

The main topic of this paper is a quantity that evaluates structural features of various statistical state distributions encoded in the same density operator ρ. It is well-known that the von Neumann entropy [1,2], defined as −ρlogρ, can evaluate how ρ deviates from a pure state, i.e., the degree of mixture of pure states. In fact, −ρlogρ can be rewritten as $\sum_{k}-\lambda_{k}\log\lambda_{k}$, where $\left\{\lambda_{k}\right\}$ are eigenvalues of ρ. It equals to zero if and only if ρ is a pure state. Note that the quantity $\sum_{k}-\lambda_{k}\log\lambda_{k}$ is the Shannon entropy for classical probability distribution $\left\{\lambda_{k}\right\}$. Thus, the von Neumann entropy evaluates only the classical distribution encoded in ρ, but not non-classical ones.

In this paper, we define a quantity such that more detailed information about the structure of statistical state distributions, especially non-classical ones, is reflected. Our discussion is fundamental, but straightforward. First, in Section 3, we mention the *“differentiation phenomenon”* which an ensemble of pure states experiences under a quantum measurement of some physical observable, say X. Each pure state is stochastically differentiated into an eigenstate of *X*. If pure states in the statistical ensemble are different, the expectation values of *X* estimated from each of them are also generally different. In Section 4, we focus on dispersion of these expectation values and discuss its mathematical property reflecting structural features of the state distribution. Finally, in Section 5, we define a *“state entropy”* (see Equation (17)). This quantity evaluates *the “diversity” of pure states constituting an ensemble.* It is proportional to the number of pure states and inversely proportional to similarities among them.

We also point to the interrelation between the state entropy and the von Neumann entropy. It can be briefly described in the following way. If a state distribution, which is encoded in ρ, is classical, then its state entropy is equal to the von Neumann entropy. The state entropies of non-classical state distributions do not exceed the latter; see the inequality of Equation (18): The state entropy is a generalization of von Neumann entropy which is extensively used in different types of quantum entropies, e.g., conditional, relative and mutual entropies [3,4,5].

State entropy evaluates non-classical statistical state distributions. To stress significance of the notion of state entropy, we explain the theoretical context of state distributions. We note that classical state distributions are always identified *after* completion of quantum measurements. Therefore, non-classical distributions may exist at the stages *before* measurements are completed, more generally, in the process of differentiation.

In Section 6, we focus on the model of differentiation that was discussed in Reference [6]. This model describes accumulation of very small state transitions experienced by the system, and each transition is mathematically represented by a map in the state space, i.e., by a “quantum channel” in the terminology of quantum information theory. A quantum channel denoted by $\Lambda^{*}$ is given by Equation (28), which is concerned with “environmental elements” around the system. They are weakly interacting with the system causing numerous small state transitions step by step, if differentiations of states occur sequentially. The above picture corresponds to an ideal “open quantum system dynamics”. To describe the process of differentiation in the system, we consider a more complicated model, assuming differentiations not only of the system state, but also in the elements of the environment. The differentiation in each environmental element is similar to the determination of a “pointer basis” in the theory of quantum decoherence proposed by Zurek [7]. In our approach, the *Lindblad equation* [8,9], which is a traditional way to describe open quantum system dynamics, is not employed directly.

We believe that the described model can be applicable to a variety of natural and mental phenomena (not only in the micro-world). The process of creation of a diversity of states in an ensemble of systems, which were originally prepared in the same pure state Ψ, through mutual interaction with environmental factors is universal. Originally, the formalism of quantum theory was established to describe microscopic phenomena, but now it is widely used in psychology, decision making, and finance (see [10,11,12,13,14,15,16,17,18,19,20,21,22,23,24,25,26,27,28,29,30,31,32,33,34,35,36,37,38]). It is also applied to model behavior of biological systems, especially the functioning of genetic and epigenetic systems (see [39,40,41,42,43,44,45,46]). We plan to explore the novel mathematical apparatus developed in this paper (based on the state entropy) for such applications elsewhere.

In psychology, there has been extensive interest in employing classical entropy for quantifying uncertainty, e.g., in decision making (entropy minimization was used to model decision biases in [47]), categorization (as a way to formalize intuitions in spontaneous grouping [48]), and learning [49,50]. We plan to apply the apparatus of the quantum state entropy to these problems.

**Figure 1:**
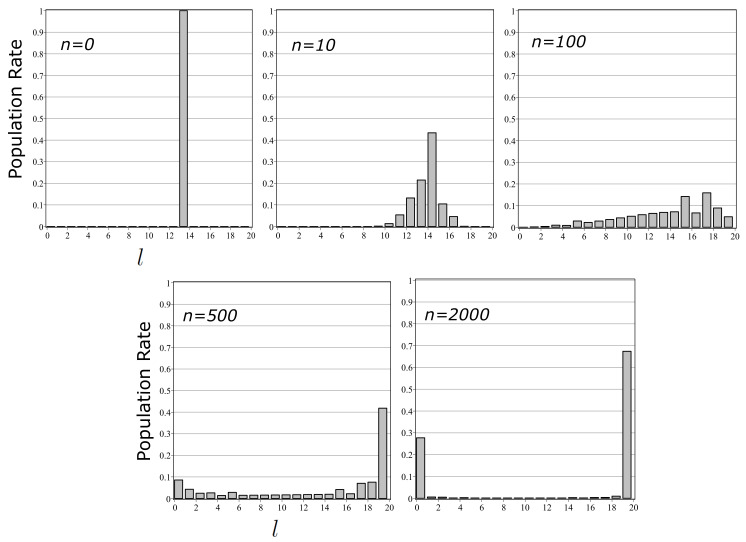
Histograms of population rates of states with $\frac{l-1}{20}<{\left|\langle \psi_{1}|\Psi_{\left\{i_{1},i_{2},\cdots ,i_{n}\right\}}\rangle \right|}^{2}\leq \frac{l}{20}\;\left(l=1,2,\cdots ,20\right)$ in the case of n=0,10,100,500 and 2000. The parameters are set by M=L=2, $|\Psi \rangle =\sqrt{0.7}|\psi_{1}\rangle +\sqrt{0.3}|\psi_{2}\rangle$ ($P_{1}=0.7,\;P_{2}=0.3$), $\nu_{1|1}=\sqrt{0.5}\;\left(\nu_{2|1}=\sqrt{0.5}\right)$ and $\nu_{1|2}=\sqrt{0.45}\;\left(\nu_{2|2}=\sqrt{0.55}\right)$. If $|\Psi_{\left\{i_{1},i_{2},\cdots ,i_{n}\right\}}\rangle \approx |\psi_{1}\rangle \left(|\psi_{2}\rangle \right)$, $|\langle \psi_{1}|\Psi_{\left\{i_{1},i_{2},\cdots ,i_{n}\right\}}\rangle {|}^{2}$ takes a value nearby 1(0). With increasing *n*, the state distribution approaches to $\left\{\left\{\psi_{1},\psi_{2}\right\},\left\{0.7,0.3\right\}\right\}$.

**Figure 2:**
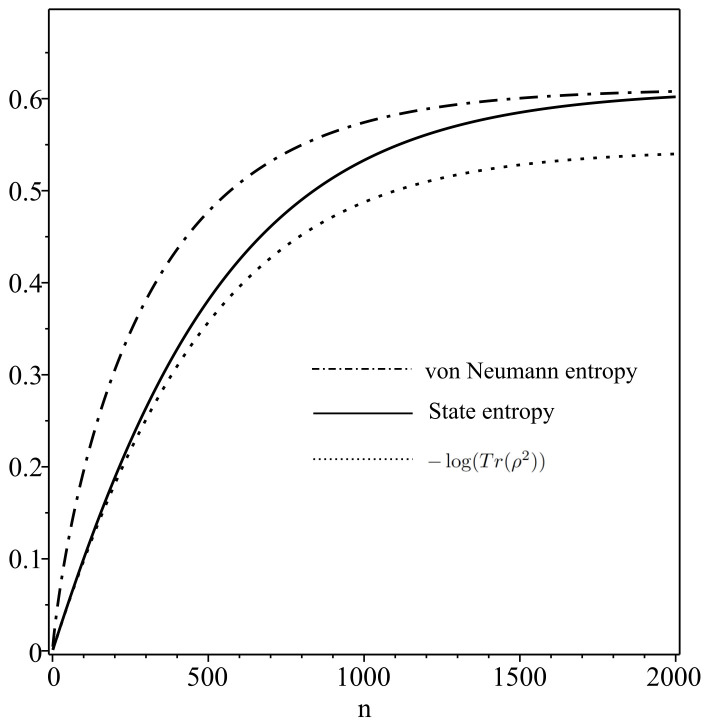
Behaviors of state entropy, von Neumann entropy and $-\log(Tr\left(\rho^{2}\right))$ at the parameters of M=L=2, $|\Psi \rangle =\sqrt{0.7}|\psi_{1}\rangle +\sqrt{0.3}|\psi_{2}\rangle$ ($P_{1}=0.7,\;P_{2}=0.3$), $\nu_{1|1}=\sqrt{0.5}\;\left(\nu_{2|1}=\sqrt{0.5}\right)$ and $\nu_{1|2}=\sqrt{0.45}\;\left(\nu_{2|2}=\sqrt{0.55}\right)$.

**Figure 3:**
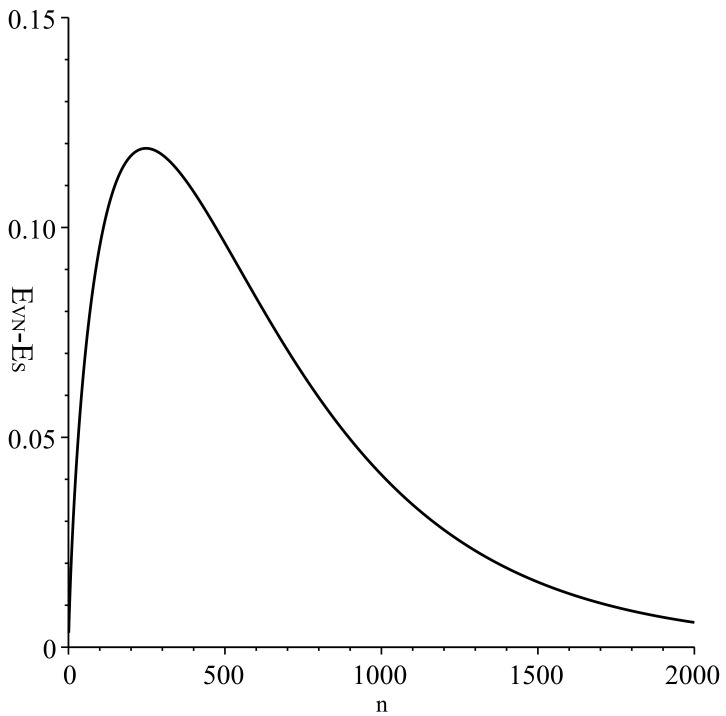
Difference between von Neumann entropy and state entropy.

As shown in Figure 1, the accumulation of transitions generated by channel $\Lambda^{*}$ represents an ideal differentiation process realized in the system. Further, in this modeling, non-classical state distributions in the intermediate stages are identified (see Equations (29)–(31)). We analyze them by means of the state entropy (see Figure 2 and Figure 3).

## 2. State Representation by Density Operator

If a physical quantity *X* is measurable in a system, the frequency probabilities {P(x)} for observed values {x} may be estimated. Then, the quantity *X* is a “stochastic variable” in terms of probability theory, and the distribution {P(x)} is a “state of the system” which can be analyzed, e.g., by calculating the expectation value E(X) or dispersion $V\left(X\right)=E\left(X^{2}\right)-{\left(E\left(X\right)\right)}^{2}$, as is usual in statistics.

The mathematical framework of quantum theory includes probability theory, where classical concepts of stochastic variables and probability distribution are expanded using the notion of “operator”. Firstly, a physical quantity is defined in the form of

(1) $$X=\sum_{k=1}^{M}x_{k}|x_{k}\rangle \langle x_{k}|.$$

This is a Hermitian operator in Hilbert space $H=C^{M}$ with real eigenvalues $x_{k}\in R(k=1,2,\cdots ,M)$ and eigenvectors $\left\{|x_{k}\rangle \right\}$. (A vector $|x\rangle \in H$ whose norm is 1 is called ket-vector, and $\langle x|$, which is Hermitian conjugate of $|x\rangle$, i.e., ${|x\rangle }^{†}=\langle x|$, is called bra-vector.) The form of Equation (1) implies that after a non-degenerate value $x_{k}$ is observed, the system under the measurement has the definite (pure) state represented by the operator $|x_{k}\rangle \langle x_{k}|$. Note that the trace of product of *X* and $|x_{k}\rangle \langle x_{k}|$ is equal to $x_{k}$;

$$Tr\left(X|x_{k}\rangle \langle x_{k}|\right)=\langle x_{k}|X|x_{k}\rangle =x_{k}.$$

For the calculation, the orthogonality of vectors, i.e., $\langle x_{k}|x_{k^{\prime }}\rangle =0$ if $k\neq k^{\prime }$, is used. Next, using the pure states $\left\{|x_{k}\rangle \langle x_{k}|\right\}$, let us construct the operator:

(2) $$\rho =\sum_{K=1}^{M}P\left(x_{k}\right)|x_{k}\rangle \langle x_{k}|.$$

where $\left\{P\left(x_{k}\right)\right\}$ corresponds to the frequency probabilities of the observed values $\left\{x_{k}\right\}$, and, in fact, the trace of Xρ is equal to the expected value E(X);

(3) Tr(Xρ)=E(X).

Mathematically, ρ is a Hermitian matrix satisfying Tr(ρ)=1 and $\langle x|\rho |x\rangle \geq 0,\;\forall x\in H=C^{M}$. Such operator is called density operator and used for representing a statistical mixture of pure states (a mixed state). A density operator may be given in the form of Schatten decomposition, i.e., represented as a diagonal matrix:

(4) $$\sum_{k=1}^{M}\lambda_{k}|\phi_{k}\rangle \langle \phi_{k}|,$$

where $\left\{\lambda_{k}\geq 0\right\}$ are the eigenvalues of the matrix (the same as probabilities $\left\{P\left(x_{k}\right)\right\}$ of ρ), and $\left\{|\phi_{k}\rangle \in H=C^{M}\right\}$ are the corresponding eigenvectors (the same as $|x_{k}\rangle$ of ρ). From Equation (4), one can obtain a picture of statistical mixture of $\left\{|\phi_{k}\rangle \langle \phi_{k}|\right\}$. (This mixture is denoted by $\left\{\phi_{k},\lambda_{k}\right\}$ hereafter.) As can be seen from the construction of ρ in Equation (2), to give a Schatten decomposition is conceptually equivalent to giving a probability distribution of measurement of some physical quantity. In this sense, the state distribution $\left\{\phi_{k},\lambda_{k}\right\}$ is “classical”. We have to point out here that decomposition of density operator is not unique, generally: By considering various linear combinations of $\left\{|\phi_{k}\rangle \right\}$, one can find a set of vectors $\left\{|\Psi_{i}\rangle ,\;i=1,\cdots N\right\}$, which satisfies

(5) $$\sum_{k=1}^{M}\lambda_{k}|\phi_{k}\rangle \langle \phi_{k}|=\sum_{i=1}^{N}P_{i}|\Psi_{i}\rangle \langle \Psi_{i}|,\;\sum_{i=1}^{N}P_{i}=1.$$

Note that N≥M and the vectors $\left\{|\Psi_{i}\rangle \in H=C^{M}\right\}$ need not be orthogonal to each other, that is, they need not be eigenstates of a single physical quantity: the state distribution $\left\{\Psi_{i},P_{i}\right\}$ is “non-classical”. There exist numerous state distributions corresponding to same density operator, other than $\left\{\phi_{k},\lambda_{k}\right\}$ and $\left\{\Psi_{i},P_{i}\right\}$, and they are non-classical.

## 3. Differentiation Phenomenon in Quantum Measurement Process

As shown in Equation (3), for the density operator

$$\rho =\sum_{K=1}^{M}P\left(x_{k}\right)|x_{k}\rangle \langle x_{k}|,$$

where $\left\{|x_{k}\rangle \langle x_{k}|\right\}$ are the eigenstates of *X*, $Tr\left(X\rho \right)=\sum_{k=1}^{M}P\left(x_{k}\right)x_{k}=E\left(X\right)$ is satisfied. In this section, noting the non-uniqueness of decomposition of density operator, we mention the meaning of Tr(Xρ) that has not been discussed in the classical theory. Let us consider a different decomposition, $\rho =\sum_{i=1}^{N}P_{i}|\Psi_{i}\rangle \langle \Psi_{i}|$, that is, we assume the existence of non-classical state distribution $\left\{\Psi_{i},P_{i}\right\}$. Then, Tr(Xρ) is described as the statistical average of the averages $\left\{{\langle X\rangle }_{\Psi_{i}}=Tr\left(X|\Psi_{i}\rangle \langle \Psi_{i}|\right)\right\}$ of observable *X* with respect to the pure states $\{\Psi_{i}\}:$

(6) $$Tr\left(X\rho \right)=\sum_{i=1}^{N}P_{i}{\langle X\rangle }_{\Psi_{i}}.$$

Each term, e.g., ${\langle X\rangle }_{\Psi }$, in the above is expanded as

(7) $${\langle X\rangle }_{\Psi }=\sum_{k=1}^{M}x_{k}{\left|\langle \Psi |x_{k}\rangle \right|}^{2}.$$

($\sum_{k=1}^{M}{\left|\langle \Psi |x_{k}\rangle \right|}^{2}=1$ is satisfied.) The square of inner product $|\langle \Psi |x_{k}\rangle {|}^{2}$ is frequently called “transition probability”. It is related to a problem of measurement that has been discussed in the quantum theory. In the concept of quantum measurement, the existence of the measurement device is considered first, because it is assumed that some interaction between the device and the system realizes the measurement of a physical quantity. Due to the interaction, the initial state of system $|\Psi \rangle \langle \Psi |$ is transferred to one of $\left\{|x_{k}\rangle \langle x_{k}|\right\}$, and the values of $\left\{x_{k}\right\}$ can be read out from the device. If ${\langle X\rangle }_{\Psi }=\sum_{k=1}^{M}x_{k}{\left|\langle \Psi |x_{k}\rangle \right|}^{2}$ means the average of outputs, the value of $|\langle \Psi |x_{k}\rangle {|}^{2}$ corresponds to the probability of transition from $|\Psi \rangle \langle \Psi |$ to $|x_{k}\rangle \langle x_{k}|$.

We interpret the process of quantum measurement as a sort of “differentiation”, in which a group of systems in one initial state is divided into groups having different states by means of external or environmental factors. The expected value of ${\langle X\rangle }_{\Psi_{i}}$ comes from one differentiation denoted by $\Psi_{i}\to \left\{x_{k}\right\}$, and the value of $Tr\left(X\rho \right)=\sum_{i=1}^{N}P_{i}{\langle X\rangle }_{\Psi_{i}}$ is to be calculated supposing a statistical mixture of *M* kinds of differentiations, $\left\{\Psi_{i}\to \left\{x_{k}\right\}\right\}\left(i=1,\cdots ,M\right)$.

## 4. Characteristic Quantity of State Distribution

We assume a definitive state distribution denoted by $\left\{\Psi_{i},P_{i}\right\}$ is given, and the calculations of $\left\{{\langle X\rangle }_{\Psi_{i}}\right\}$ are possible. The average of $\left\{{\langle X\rangle }_{\Psi_{i}}\right\}$, i.e., $\sum_{i=1}^{N}P_{i}{\langle X\rangle }_{\Psi_{i}}=Tr\left(X\rho \right)$ depends only on the density operator ρ, in which $\left\{\Psi_{i},P_{i}\right\}$ is encoded. A statistical quantity reflecting more detailed information on the structure of $\left\{\Psi_{i},P_{i}\right\}$ is dispersion of $\{{\langle X\rangle }_{\Psi_{i}}\},$ formulated as

(8) $$V\left(\left\{{\langle X\rangle }_{\Psi_{i}},P_{i}\right\}\right)=\sum_{i=1}^{N}P_{k}{\langle X\rangle }_{\Psi_{i}}^{2}-{\left(\sum_{i=1}^{N}P_{i}{\langle X\rangle }_{\Psi_{i}}\right)}^{2}=\sum_{i=1}^{N}P_{i}{\langle X\rangle }_{\Psi_{i}}^{2}-{\left(Tr(X\rho )\right)}^{2}.$$

Below, we prove the inequality

(9) $$V\left(\left\{x_{i},P\left(x_{i}\right)\right\}\right)\geq V\left(\left\{{\langle X\rangle }_{\Psi_{i}},P_{i}\right\}\right),$$

where $V(\left\{x_{i},P\left(x_{i}\right)\right\})$ is the dispersion of observable *X*, i.e., its dispersion with respect to the probability distribution encoded in the Shatten decomposition (see Equation (1)), corresponding to the spectral decomposition of the observable *X* (see Equation (2)). Thus, the probability distribution corresponding to the spectral decomposition of *X* maximizes the dispersions with respect to decompositions in Equation (5). The inequality for dispersions can be interpreted by the theory of weak measurements. The quantities ${\langle X\rangle }_{\Psi_{i}}$ can be interpreted as weak values. In this framework, the inequality in Equation (9) simply means that dispersion of a weak measurement is always majorized by dispersion of the “maximally disturbing measurement”, represented by a Hermitian operator. At the same time, we are aware that interpretation of weak values is a complex foundational problem of itself.

To prove the inequality in Equation (9), let us consider the first term given by

(10) $$D\left(\left\{{\langle X\rangle }_{\Psi_{i}},P_{i}\right\}\right)=\sum_{i=1}^{N}P_{i}{\langle X\rangle }_{\Psi_{i}}^{2}.$$

Let us note the following inequality

(11) $$\sum_{k=1}^{M}\langle x_{k}|\rho |x_{k}\rangle {\left(x_{k}\right)}^{2}\geq D\left(\left\{{\langle X\rangle }_{\Psi_{i}},P_{i}\right\}\right)\geq {\left(Tr(X\rho )\right)}^{2}.$$

which follows from the convexity of $y=x^{2}$, because

(12) $$\sum_{i=1}^{N}P_{i}{\langle X\rangle }_{\Psi_{i}}^{2}\geq {\left(Tr(X\sum_{i=1}^{N}P_{i}|\Psi_{i}\rangle \langle \Psi_{i}|\right)}^{2}={\left(Tr(X\rho )\right)}^{2},$$

and since

$$\sum_{i=1}^{N}P_{i}{\langle X\rangle }_{\Psi_{i}}^{2}=\sum_{i=1}^{N}P_{i}{\left(\sum_{k=1}^{M}x_{k}{\left|\langle x_{k}|\Psi_{i}\rangle \right|}^{2}\right)}^{2}$$

where $X=\sum_{k=1}^{M}x_{k}|x_{k}\rangle \langle x_{k}|$, one can see

(13) $$\sum_{i=1}^{N}P_{i}{\langle X\rangle }_{\Psi_{i}}^{2}\leq \sum_{i=1}^{N}\sum_{k=1}^{M}P_{i}{\left|\langle x_{k}|\Psi_{i}\rangle \right|}^{2}{\left(x_{k}\right)}^{2}=\sum_{k=1}^{M}\langle x_{k}|\rho |x_{k}\rangle {\left(x_{k}\right)}^{2}.$$

Such inequality can be derived with the use of other convex functions, not limited to $y=x^{2}$. Even if the dispersion *V* is defined as

(14) $$V\left(\left\{{\langle X\rangle }_{\Psi_{i}},P_{i}\right\}\right)=\sum_{i=1}^{N}P_{i}f\left({\langle X\rangle }_{\Psi_{i}}\right))-f\left(Tr\left(X\rho \right)\right),$$

using another convex function, e.g., f(x), the result holds true, that is, the inequality

(15) $$\sum_{k=1}^{M}\langle x_{k}|\rho |x_{k}\rangle f\left(x_{k}\right)-f\left(Tr\left(X\rho \right)\right)\geq V\left(\left\{{\langle X\rangle }_{\Psi_{i}},P_{i}\right\}\right)\geq 0,$$

is satisfied.

We redefine the first term *D* of Equation (10) as

(16) $$D\left(\left\{{\langle X\rangle }_{\Psi_{i}},P_{i}\right\}\right)=\sum_{i=1}^{N}P_{i}f\left({\langle X\rangle }_{\Psi_{i}}\right)).$$

As discussed in the next section, we believe that, under proper choices of *X* and f(x), this *D* itself becomes a quantity that captures structural features of $\left\{\Psi_{i},P_{i}\right\}$.

## 5. State Entropy

In this section, we consider *D* of Equation (16) in the case of X=ρ and f(x)=−logx:

(17) $$D\left(\left\{{\langle \rho \rangle }_{\Psi_{i}},P_{i}\right\}\right)=-\sum_{i=1}^{N}P_{i}\log{\langle \rho \rangle }_{\Psi_{i}}.$$

Here, we fix the state distribution $\left\{\Psi_{i},P_{i}\right\}$ for the density operator ρ, and y=−logx is our choice of a convex function. What does the above *D* tell us about $\left\{\Psi_{i},P_{i}\right\}$? To discuss this question, we first focus on the term of $Tr\left(\rho |\Psi_{i}\rangle \langle \Psi_{i}|\right)={\langle \rho \rangle }_{\Psi_{i}}$. Since

$${\langle \rho \rangle }_{\Psi_{i}}=P_{i}+\sum_{j\neq i}P_{j}{\left|\langle \Psi_{i}|\Psi_{j}\rangle \right|}^{2},$$

$$1>{\langle \rho \rangle }_{\Psi_{i}}\geq P_{i},$$

is satisfied. One can see, ${\langle \rho \rangle }_{\Psi_{i}}=P_{i}$ if all the vectors $\left\{|\Psi_{j\neq i}\rangle \right\}$ are orthogonal to $|\Psi_{i}\rangle$, and ${\langle \rho \rangle }_{\Psi_{i}}=1$ if all $\left\{|\Psi_{j\neq i}\rangle \right\}$ are parallel to $|\Psi_{i}\rangle$. Based on this, we interpret ${\langle \rho \rangle }_{\Psi_{i}}$ as a degree of “similarity” of $|\Psi_{i}\rangle \langle \Psi_{i}|$ and ρ. This interpretation of quantity ${\langle \rho \rangle }_{\Psi_{i}}$ as the degree of similarity can also be illustrated by the representation of the operators $|\Psi_{i}\rangle \langle \Psi_{i}|$ and ρ as vectors in the Hilbert space of Hilbert–Schmidt operators endowed with the scalar product $\langle A|B\rangle =TrA^{☆}B.$ We start with the remark that $\langle A|B\rangle =\cos\theta_{AB}{∥A∥}_{2}{∥B∥}_{2}$, where ${∥\cdot ∥}_{2}$ is the Hilbert–Schmidt norm; we also remark that, for a self-adjoint operator ${A,∥A∥}_{2}=TrA^{2}$. In particular, the norm of any pure state and the norm of any projector are equal to one. We have

$$\langle |\Psi_{i}\rangle \langle \Psi_{i}||\rho \rangle =Tr\left(\sum_{j}P_{j}|\Psi_{i}\rangle \langle \Psi_{i}\left|\right|\Psi_{j}\rangle \langle \Psi_{j}|\right)={\langle \rho \rangle }_{\Psi_{i}}.$$

Hence,

$${\langle \rho \rangle }_{\Psi_{i}}=\cos\theta \;Tr\rho^{2},$$

where θ is the angle between the vectors $|\Psi_{i}\rangle \langle \Psi_{i}|$ and ρ. The scaling coefficient $Tr\rho^{2}$ is the purity of the state ρ.

Further, noting that y=−logx is a monotonically decreasing function, we interpret $-\log{\langle \rho \rangle }_{\Psi_{i}}=-\log\cos\theta -\log Tr\rho^{2}$ as a degree of orthogonality between the vectors $|\Psi_{i}\rangle \langle \Psi_{i}|$ and ρ. We note that the following inequality is satisfied: $-\log P_{i}\geq -\log\left({\langle \rho \rangle }_{\Psi_{i}}\right)>0$.

In general, any convex and monotonically decreasing function is allowed as f(x). The average of orthogonality $-\log{\langle \rho \rangle }_{\Psi_{i}}$, i.e., $\sum_{i=1}^{N}P_{i}\left(-\log{\langle \rho \rangle }_{\Psi_{i}}\right)$ corresponds to *D* of Equation (17). Generally, the value of *D* will increase in proportion to the number of states and decrease in proportion to similarities among them. That is why we call the value *D* “state diversity” or “state entropy”.

The following inequality shows the significance of state entropy *D*:

(18) $$\sum_{k=1}^{M}\lambda_{k}\left(-\log\lambda_{k}\right)\geq D\left(\left\{{\langle \rho \rangle }_{\Psi_{i}},P_{i}\right\}\right)\geq -\log\left(Tr\left(\rho^{2}\right)\right).$$

It can be derived with the use of convexity of y=−logx, in a similar way as derivation of Equation (11). In the above form, $\left\{\lambda_{k}\right\}$ are the eigenvalues of $\rho =\sum_{k=1}^{M}\lambda_{k}|\phi_{k}\rangle \langle \phi_{k}|$. The term of $\sum_{k=1}^{M}\lambda_{k}\left(-\log\lambda_{k}\right)$ in the left-hand side corresponds to von Neumann entropy given by −ρlogρ. Further, $Tr(\rho^{2})$ in the right-hand side is a well-known quantity in the quantum theory, too. The von Neumann entropy −ρlogρ and $Tr(\rho^{2})$ are frequently used to evaluate the degree of “mixing” in ρ: if ρ is pure, then, −ρlogρ=0 and $Tr(\rho^{2})=1$. If ρ is a mixed state, −ρlogρ>0 and $Tr(\rho^{2})<1$, and especially, when $\lambda_{1}=\lambda_{2}=\cdots =\lambda_{M}=1/M$, −ρlogρ takes the maximum value of logM, and $Tr(\rho^{2})$ takes minimum value of 1/M. Mathematically, these two quantities have the relation of $-\rho \log\rho \geq -\log(Tr\left(\rho^{2}\right))$. The inequality in Equation (18) implies that the intermediate values between these two correspond to other kinds of state entropy, which can estimated for various non-classical state distributions reducing to ρ. In other words, the well-known −ρlogρ and $Tr(\rho^{2})$ are newly interpreted as maximum and minimum values of state entropy.

Note that the state entropy *D* is different from the generalized quantum entropic measures that have been proposed until now. This point is mentioned in the Appendix A.

## 6. Model of Differentiation and Calculation of State Entropy

As mentioned in Section 2, a Schatten decomposition of a density operator such as Equation (2) represents a probabilistic distribution of orthogonal pure states. Such an ensemble of states is postulated to be the resulting state of the system after measurement of some physical quantity, whose eigenstates are orthogonal. On the other hand, using another decomposition of the density operator, a mixture of non-orthogonal pure states may be obtained, and we call such mixture non-classical. In Section 3, we point out that the essence of quantum measurement is state differentiation caused by external or environmental factors. If state distribution corresponding to Schatten decomposition is a goal of differentiation, various non-classical ones will appear in intermediate stages before reaching the goal. Below, we model this mechanism as proposed in [6]. This model mathematically explains what state distribution may occur in the differentiation process. Our aim in this section is to evaluate state structural features by using the state entropy defined in Section 5.

Let us consider a typical state transition caused by a quantum measurement, which is denoted by

$$\Psi \to \{\psi_{k},P_{k}\}.$$

Ψ means an initial state of system represented by $|\Psi \rangle \langle \Psi |$, and $\left\{\psi_{k},P_{k}\right\}$ means a distribution where the states $\left\{|\psi_{k}\rangle \langle \psi_{k}|\right\}$ exist with probabilities $\left\{P_{k}\right\}$. $\left\{|\psi_{k}\rangle \right\}$ correspond to eigenstates of some physical quantity defined in Hibert space $H=C^{M}$, and the initial vector $|\Psi \rangle$ is expanded as

$$|\Psi \rangle =\sum_{k=1}^{M}\sqrt{P_{k}}|\psi_{k}\rangle ,$$

where $\sqrt{P_{k}}$ means a complex number satisfying $|\sqrt{P_{k}}{|}^{2}=P_{k}$, that is,

(19) $$|\Psi \rangle \langle \Psi |=\sum_{k=1}^{M}P_{k}|\psi_{k}\rangle \langle \psi_{k}|+\sum_{k\neq k^{\prime }}\sqrt{P_{k}}{\sqrt{P_{k^{\prime }}}}^{*}|\psi_{k}\rangle \langle \psi_{k^{\prime }}|.$$

The first term $\sum_{k=1}^{M}P_{k}|\psi_{k}\rangle \langle \psi_{k}|$ corresponds to the distribution $\left\{\psi_{k},P_{k}\right\}$, and, therefore, vanishing of the second term, the process called “decoherence” in quantum theory, means accomplishment of the measurement. The relation of Ψ and $\left\{\psi_{k},P_{k}\right\}$ is represented as

(20) $$\sum_{k=1}^{M}M_{k}|\Psi \rangle \langle \Psi |M_{k}^{*}=\sum_{k=1}^{M}{\left|\langle \psi_{k}|\Psi \rangle \right|}^{2}|\psi_{k}\rangle \langle \psi_{k}|=\sum_{k=1}^{M}P_{k}|\psi_{k}\rangle \langle \psi_{k}|,$$

with the use of projection operator $M_{k}=|\psi_{k}\rangle \langle \psi_{k}|$. (The transition probability $|\langle \psi_{k}|\Psi \rangle {|}^{2}$ is equal to $P_{k}$.)

If the above transition is interpreted as a sort of differentiation, its development, i.e., what state distributions occur between Ψ and $\left\{\psi_{k},P_{k}\right\}$, becomes a crucial concern. The model of differentiation, which was proposed in [6], presents the picture that the initial state Ψ is differentiated to $\left\{\psi_{k},P_{k}\right\}$ step by step through many state transitions. Each state transition is described with use of a map from state to state, which is denoted by $\Lambda^{*}$. The map is called “quantum channel” in quantum information theory. A chain of state transitions given as

$$\rho \left(0\right)=|\Psi \rangle \langle \Psi |\to \rho \left(1\right)=\Lambda^{*}\left(\rho \left(0\right)\right)\to \rho \left(2\right)=\Lambda^{*}\left(\rho \left(1\right)\right)\to \cdots \to \rho \left(n\right)=\Lambda^{*}\left(\rho \left(n-1\right)\right),$$

is regarded as a process of differentiation, if

(21) $$\underset{n\to \infty }{\mathrm{lin}}\rho \left(n\right)=\sum_{k=1}^{M}P_{k}|\psi_{k}\rangle \langle \psi_{k}|,$$

is satisfied. A channel $\Lambda^{*}$ is to be defined based on the following: There exist numerous environmental elements around the system. Initially, states of system and these elements are given independently. Let $|\Phi \rangle \langle \Phi |$ be the initial state of one element, which is defined on a space $K_{1}=C^{L}$. The initial compound state of the system and the element, on the space $H⊗K_{1}$, is factorized

$$|\Psi \rangle \langle \Psi |⊗|\Phi \rangle \langle \Phi |.$$

At the next step, the states of the system and the element become non-separable. Such compound state is generally defined as

$$U|\Psi \rangle \langle \Psi |⊗|\Phi \rangle \langle \Phi |U^{*},$$

using a unitary operator *U* on $H⊗K_{1}$. The unitary transformation *U* specifies a correlation generated between the system and the element, and, in the modeling, the following form is assumed:

(22) $$U=\sum_{k=1}^{M}|\psi_{k}\rangle \langle \psi_{k}|⊗u_{k}.$$

where $u_{k}$ is a unitary on $K_{1}$. Actually, by this *U*, the vector $|\Psi \rangle ⊗|\Phi \rangle$ is transformed to

$$U|\Psi \rangle ⊗|\Phi \rangle =\sum_{k=1}^{M}\sqrt{P_{k}}|\psi_{k}\rangle ⊗|\Phi_{k}\rangle ,$$

where $|\Phi_{k}\rangle =u_{k}|\Phi \rangle$. Then, the states of the system and the element are “entangled”, since the above form cannot be factorized into two vectors independently defined on H and $K_{1}$, if $|\Phi_{k}\rangle \neq |\Phi_{k^{\prime }}\rangle$ for some $k\neq k^{\prime }$. A compound state at the third step is described as

(23) $$\sum_{j=1}^{L}\left(I⊗{\bar{M}}_{j}\right)U|\Psi \rangle \langle \Psi |⊗|\Phi \rangle \langle \Phi |U^{*}{\left(I⊗{\bar{M}}_{j}\right)}^{*}.$$

$\left\{{\bar{M}}_{j}\right\}$ are projection operators corresponding to the basis set of $K_{1}=C^{L}$, say $\left\{|\phi_{j}\rangle \right\}$. As can be seen from Equation (20), a state transition given by a projection operator mathematically represents accomplishment of differentiation. The operation of $\left\{{\bar{M}}_{j}\right\}$ means that the state of the element is eventually differentiated into $\left\{|\phi_{j}\rangle \langle \phi_{j}|\right\}$. Note that the states of the system and the element are correlated at the second step. Thus, the state of the system is affected by the differentiation. Actually, Equation (23) may be rewritten to

(24) $$\sum_{j=1}^{L}E_{j}|\Psi \rangle \langle \Psi |E_{j}^{*}⊗|\phi_{j}\rangle \langle \phi_{j}|,$$

by introducing the operator,

(25) $$E_{j}=\sum_{k=1}^{M}\langle \phi_{j}|\Phi_{k}\rangle |\psi_{k}\rangle \langle \psi_{k}|=\sum_{k=1}^{M}\nu_{j|k}|\psi_{k}\rangle \langle \psi_{k}|.$$

The above form implies that the state of the element of the environment gets transformed to $|\phi_{j}\rangle \langle \phi_{j}|$ with probability,

(26) $$P_{j}=Tr\left(E_{j}|\Psi \rangle \langle \Psi |E_{j}^{*}⊗|\phi_{j}\rangle \langle \phi_{j}|\right)=\langle \Psi |E_{j}^{*}E_{j}|\Psi \rangle ,$$

and at the same time the state of the system transits to

(27) $$|\Psi_{j}\rangle \langle \Psi_{j}|=\frac{1}{P_{j}}E_{j}|\Psi \rangle \langle \Psi |E_{j}^{*}.$$

The operator $E_{j}$ introduced in Equation (25) is called Kraus operator and satisfies $\sum_{j=1}^{L}E_{j}^{*}E_{j}=I$. (In general, a set of Hermitian positive operators $\left\{F_{i}\right\}$ with $\sum_{i=1}^{N}F_{i}=I$ is called positive-operator valued measure (POVM).) With the use of $\{E_{j}\},$ a quantum channel $\Lambda^{*}$ is defined:

(28) $$\Lambda^{*}\left(\cdot \right)=\sum_{j=1}^{L}E_{j}\cdot E_{j}^{*}.$$

$\Lambda^{*}\left(|\Psi \rangle \langle \Psi |\right)=\sum_{j=1}^{L}E_{j}|\Psi \rangle \langle \Psi |E_{j}^{*}$ means the density operator obtained from the partial trace of the compound state, $Tr_{K}\left(\sum_{j=1}^{L}E_{j}|\Psi \rangle \langle \Psi |E_{j}^{*}⊗|\phi_{j}\rangle \langle \phi_{j}|\right)$.

The other environmental elements are defined in Hilbert spaces denoted by $K_{2,3\cdots }$. If they interact with the system in a similar way,

$$\rho \left(n\right)=\Lambda^{*}\left(\rho \left(n-1\right)\right)=\cdots =\Lambda^{*}\left(\Lambda^{*}\left(\cdots \Lambda^{*}\left(|\Psi \rangle \langle \Psi |\right)\cdots \right)\right),$$

is defined as the density operator of the system that is obtained after interacting with *n* environmental elements. From the definition of $\Lambda^{*}$ (see Equation (28)), this ρ(n) is decomposed as

(29) $$\rho \left(n\right)=\sum_{\left\{j_{1},j_{2},\cdots ,j_{n}\right\}}P_{\left\{j_{1},j_{2},\cdots ,j_{n}\right\}}|\Psi_{\left\{j_{1},j_{2},\cdots ,j_{n}\right\}}\rangle \langle \Psi_{\left\{j_{1},j_{2},\cdots ,j_{n}\right\}}|,$$

where

(30) $$P_{\left\{j_{1},j_{2},\cdots ,j_{n}\right\}}=\langle \Psi |E_{\left\{j_{1},j_{2},\cdots ,j_{n}\right\}}^{*}E_{\left\{j_{1},j_{2},\cdots ,j_{n}\right\}}|\Psi \rangle ,$$

and

(31) $$|\Psi_{\left\{j_{1},j_{2},\cdots ,j_{n}\right\}}\rangle =\frac{1}{P_{\left\{j_{1},j_{2},\cdots ,j_{n}\right\}}}E_{\left\{j_{1},j_{2},\cdots ,j_{n}\right\}}|\Psi \rangle .$$

(The notation $E_{\left\{j_{1},j_{2},\cdots ,j_{n}\right\}}$ means $E_{j_{n}}\cdots E_{j_{2}}E_{j_{1}}$.) $P_{\left\{j_{1},j_{2},\cdots ,j_{n}\right\}}$ is the probability that the states of *n* environmental elements eventually become $\left\{\phi_{j_{1}},\phi_{j_{2}},\cdots ,\phi_{j_{n}}\right\}$, and $|\Psi_{\left\{j_{1},j_{2},\cdots ,j_{n}\right\}}\rangle \langle \Psi_{\left\{j_{1},j_{2},\cdots ,j_{n}\right\}}|$ is a pure state of the system at this event. It should be noted here that the density operator ρ(n) can be expanded as

(32) $$\rho \left(n\right)=\sum_{k=1}^{M}P_{k}|\psi_{k}\rangle \langle \psi_{k}|+\sum_{k\neq k^{\prime }}\sqrt{P_{k}}{\sqrt{P_{k^{\prime }}}}^{*}{\left(\langle \Phi_{k}|\Phi_{k^{\prime }}\rangle \right)}^{n}|\psi_{k}\rangle \langle \psi_{k^{\prime }}|,$$

by using Equation (19) and the property of

$$\Lambda^{*}\left(|\psi_{k}\rangle \langle \psi_{k}^{\prime }|\right)=\sum_{j=1}^{L}\langle \Phi_{k}|\psi_{j}\rangle \langle \psi_{j}|\Phi_{k^{\prime }}\rangle |\psi_{k}\rangle \langle \psi_{k}^{\prime }|=\langle \Phi_{k}|\Phi_{k^{\prime }}\rangle |\psi_{k}\rangle \langle \psi_{k}^{\prime }|.$$

Since $|\langle \Phi_{k}|\Phi_{k^{\prime }}\rangle |<1$, the condition of Equation (21), i.e., ${\mathrm{lin}}_{n\to \infty }\rho \left(n\right)=\sum_{k=1}^{M}P_{k}|\psi_{k}\rangle \langle \psi_{k}|$. is clearly satisfied. Thus, the state distribution $\left\{\Psi_{\left\{j_{1},j_{2},\cdots ,j_{n}\right\}},P_{\left\{j_{1},j_{2},\cdots ,j_{n}\right\}}\right\}$, which is encoded in ρ(n), is identified at an intermediate stage in the differentiation process $\Psi \to \{\psi_{k},P_{k}\}$.

Figure 1 shows the result of computational simulation with M=L=2, $|\Psi \rangle =\sqrt{0.7}|\psi_{1}\rangle +\sqrt{0.3}|\psi_{2}\rangle$ ($P_{1}=0.7,\;P_{2}=0.3$), $\nu_{1|1}=\sqrt{0.5}\;\left(\nu_{2|1}=\sqrt{0.5}\right)$ and $\nu_{1|2}=\sqrt{0.45}\;\left(\nu_{2|2}=\sqrt{0.55}\right)$. The histograms of population rates of states with $\frac{l-1}{20}<{\left|\langle \psi_{1}|\Psi_{\left\{j_{1},j_{2},\cdots ,j_{n}\right\}}\rangle \right|}^{2}\leq \frac{l}{20}\;\left(l=1,2,\cdots ,20\right)$ are calculated in the case of n=0,10,100,500 and 2000. One can see that with increasing *n*, the state distribution approaches the goal of differentiation, i.e., $\left\{\left\{\psi_{1},\psi_{2}\right\},\left\{0.7,0.3\right\}\right\}$.

Figure 2 shows the behavior of the state entropy *D*, von Neumann entropy and $-\log(Tr\left(\rho^{2}\right))$ for the distribution $\left\{\Psi_{\left\{i_{1},i_{2},\cdots ,i_{n}\right\}},P_{\left\{i_{1},i_{2},\cdots ,i_{n}\right\}}\right\}$, which are calculated in the same setting of parameters. One can directly see that the inequality of Equation (18) is satisfied at any *n*. Note that the state entropy *D* takes values close to von Neumann entropy at very large *n*. In fact, as shown in Figure 3, the difference between von Neumann entropy and the state entropy is noticeable mostly at earlier stages. These results imply that state distributions appearing in the differentiation process are non-classical in general.

## 7. Conclusions

The state entropy is a truly non-classical quantity because it depends not only on statistical probabilities, but also on similarities among states. The differentiation phenomenon is also non-classical, because it is interpreted as dynamics of the probabilities and similarities. Definition of the state entropy and modeling of the differentiation process are impossible in the framework of classical probability theory.

We believe that evaluation of an ensemble of systems by the state entropy fits the empirical reasoning: No matter how many systems are in the ensemble, we may not recognize high diversity if we know that these states are not very different. Further, we believe that various areas of the nature dynamics of character change in the population of individuals is very much like the differentiation phenomena. This makes prospects of the quantum-like formalism grow stronger.

## Appendix A. State Entropy and other Quantum Entropies

In this paper, we propose the state entropy,

$$D\left(\left\{{\langle \rho \rangle }_{\Psi_{i}},P_{i}\right\}\right)=\sum_{i=1}^{N}P_{i}\left(-\log\left(Tr\left(\rho |\Psi_{i}\rangle \langle \Psi_{i}|\right)\right)\right).$$

More generally, it is defined as

$$D\left(\left\{{\langle \rho \rangle }_{\Psi_{i}},P_{i}\right\}\right)=\sum_{i=1}^{N}P_{i}f\left(Tr\left(\rho |\Psi_{i}\rangle \langle \Psi_{i}|\right)\right),$$

by using a convex and monotone decreasing function f(x). Mathematically, $D(\left\{{\langle \rho \rangle }_{\Psi_{i}},P_{i}\right\}$ depends on the way of decomposition of ρ. As discussed in Section 5, for $\rho =\sum_{i=1}^{N}P_{i}|\Psi_{i}\rangle \langle \Psi_{i}|$, $f\left(Tr\left(\rho |\Psi_{i}\rangle \langle \Psi_{i}|\right)\right)=f\left({\langle \rho \rangle }_{\Psi_{i}}\right)$ is interpreted as the degree of orthogonality between $|\Psi_{i}\rangle \langle \Psi_{i}|$ and ρ. In this sense, *D* evaluates a sort of diversity in the state distribution $\left\{\Psi_{i},P_{i}\right\}$, and it takes the maximal value equivalent to −Tr(ρlogρ) for the Schatten decomposition (see Equation (18)).

On the other hand, there are many mathematical expansions of von Neumann entropy. As examples, the quantum version of Rényi entropy [51],

(A1) $$R_{\alpha }\left(\rho \right)=\frac{1}{1-\alpha }\log\left(Tr\left(\rho^{\alpha }\right)\right),$$

and the one of Tsallis entropy [52],

(A2) $$T_{\alpha }\left(\rho \right)=\frac{1}{1-\alpha }\left(Tr\left(\rho^{\alpha }\right)-1\right),$$

are well-known. These entropies approach to von Neumann entropy S(ρ)=−Tr(ρlogρ) in the limit α→1. The index α, which is called the entropic parameter, is nonnegative and α≠1. Further, such generalized entropies are uniformly represented in the form of quantum version of Salicrú entropy [53,54], which is given by

(A3) $$H_{\left(h,\phi \right)}\left(\rho \right)=h\left(Tr\phi \left(\rho \right)\right),$$

where the functions h:R→R and ϕ:[0,1]→R satisfy either of the following conditions: (i) *h* is increasing and ϕ is concave; or (ii) *h* is decreasing and ϕ is convex. In the form of Rényi entropy, $h\left(x\right)=\frac{\log(x)}{1-\alpha }$ and $\phi \left(x\right)=x^{\alpha }$, and in the form of Tsallis entropy, $h\left(x\right)=\frac{x-1}{1-\alpha }$ and $\phi \left(x\right)=x^{\alpha }$. Of course, von Neumann entropy is also recovered at h(x)=x and ϕ(x)=−xlogx.

Here, we have to point out that $H_{\left(h,\phi \right)}\left(\rho \right)$ is practically calculated as

$$H_{\left(h,\phi \right)}\left(\rho \right)=h\left(\sum_{k=1}^{M}\phi \left(\lambda_{k}\right)\right),$$

by using the eigenvalues of ρ, that is, any quantum entropic measure that is reduced into $H_{\left(h,\phi \right)}\left(\rho \right)$ does not depend on the way of decomposition of ρ. The state entropy is different in this point. Actually, it is clear that $D(\left\{{\langle \rho \rangle }_{\Psi_{i}},P_{i}\right\})$ is not recovered in the form of $H_{\left(h,\phi \right)}\left(\rho \right)$.
